# Dissipative Particle Dynamics Simulations for Phospholipid Membranes Based on a Four-To-One Coarse-Grained Mapping Scheme

**DOI:** 10.1371/journal.pone.0154568

**Published:** 2016-05-03

**Authors:** Xiaoxu Li, Lianghui Gao, Weihai Fang

**Affiliations:** Key Laboratory of Theoretical and Computational Photochemistry, Ministry of Education, College of Chemistry, Beijing Normal University, Beijing 100875, China; Hong Kong University of Science and Technology, HONG KONG

## Abstract

In this article, a new set of parameters compatible with the dissipative particle dynamics (DPD) force field is developed for phospholipids. The coarse-grained (CG) models of these molecules are constructed by mapping four heavy atoms and their attached hydrogen atoms to one bead. The beads are divided into types distinguished by charge type, polarizability, and hydrogen-bonding capacity. First, we derive the relationship between the DPD repulsive force and Flory-Huggins *χ*-parameters based on this four-to-one CG mapping scheme. Then, we optimize the DPD force parameters for phospholipids. The feasibility of this model is demonstrated by simulating the structural and thermodynamic properties of lipid bilayer membranes, including the membrane thickness, the area per lipid, the lipid tail orientation, the bending rigidity, the rupture behavior, and the potential of mean force for lipid flip-flop.

## Introduction

Molecular modeling is an important tool for investigating the assembly of biomolecules in condensed phases and solutions. Computer simulations can provide detailed information to elucidate how small changes in local conformation propagate to affect the properties of macromolecular complexes. In principle, the widely used all-atom molecular dynamics (AAMD) simulation can provide insight into the links between structures and physical properties [1]. However, the assembly of complex biomolecular systems usually spans length and time scales far beyond the molecular scales [2, 3]. To handle the entire perspective of phenomena in complex materials, various coarse-grained (CG) methods, including coarse-grained molecular dynamics (CGMD) [3–7] and dissipative particle dynamics (DPD) simulations [8–25], have been developed.

In CG modeling, the starting point is to group a few atoms into one quasiparticle (or bead) by averaging some set of unessential degrees of freedom [2, 3]. The CG force field is then adjusted to capture the most fundamental physical and chemical properties of the system [7, 14, 19, 21, 23, 26–28]. In this way, CG simulations are able to reach biologically relevant time and length scales and interpret experimental data. Among the CG methods, the MARTINI model [4–6, 29] is a very popular model that is based on a four-to-one mapping scheme, i.e., it combines four heavy atoms and their associated hydrogen atoms into a single CG site. The MARTINI force field (including bonded and non-bonded potentials) is parameterized to match the structure and partitioning free-energy obtained from all-atom simulations or experimental data. Another widely used CG model is the DPD method. The DPD model combines atoms into soft beads that interact via explicit soft conservative, random, and dissipative forces [8–12]. The non-bonded DPD force field is derived by linking the force parameters to the *χ*-parameters in Flory-Huggins theory. Compared to most CGMD models, the soft potentials used in DPD method sufficiently speed up the simulation. More importantly, the hydrodynamic behavior and the effects of dissipation and thermal fluctuation are accurately included in the DPD model. As a result, with the proper force field, DPD can bridge the microscopic and macroscopic level with consistent length and time scales and thermodynamic properties [19, 30]. It thus allows for multi-scaled simulations by coupling the hydrodynamics of the particle region and the continuum region [30].

**Fig 1:**
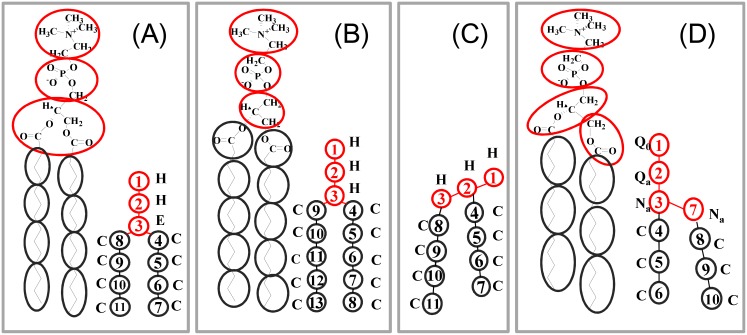
Atomic representation of DMPC lipid and its corresponding CG model. (A): Inverted Y-shape model by Groot and Rabone [10]. (B): Modified inverted Y-shape model by Kranenburg, Nicolas and Smit [23]. (C): *λ*-shape model by Shillcock and Lipowsky [12]. (D): h-shape MARTINI model used by us [31–33].

DPD has been actively applied in the study of biopolymers and self-assemble systems, such as polypeptides and lipid membranes [13–19, 21–25]. In DPD modeling, lipids are represented by polymers composed of hydrophilic and hydrophobic beads connected by bonds. The desired arrangement of the amphiphilic segments can be achieved by bonding the beads in a specific way. In the literature, a couple of DPD models have been applied to phospholipids, as shown in Fig 1. With the mapping of one water molecule on one bead, Kranenburg et al. created a CG lipid model almost identical to a united atom model [23]. Groot and Warren presented an inverted Y-shape model [10], where each lipid has three head group beads and two hydrophobic tails, Fig 1A. Kranenburg et al. regrouped the atoms and presented a modified inverted Y-shape model [23] (Fig 1B), where each lipid tail has one more hydrophobic bead compared to Fig 1A. Shilcock and Lipowsky modified the Y-shape model to a *λ*-shape model [12], Fig 1C. We recently made a DPD-versioned MARTINI-like h-shape lipid model by mapping about four heavy atoms onto one bead [31–33] (Fig 1D), where the beads were divided into types distinguished by polarizability and hydrogen-bonding capacity. The four-to-one CG mapping was also applied to sufactants and polymer brushes in DPD simulations by Neimark’s group [34–37]. Based on these mapping schemes, various sets of force fields were assigned to the CG lipids [10, 12, 14, 23, 31–33]. Even for the same CG model, such as the Y-shape model in Fig 1B, at least four force parameter sets were used in the literature [23]. Though each model, with proper force parameters, can reproduce some physically reasonable properties of the lipid membrane, the diversity of the CG mapping schemes and force fields makes it difficult to compare data and results in the published reports.

In DPD simulations, the interaction unit is represented by a bead with similar mass and volume. Meanwhile, to characterize the physical and chemical properties of biomolecules, such as charges, hydrophilicities, and polarizabilities, the molecules are usually separated into CG beads based on the functional group. According to these two rules, care must be taken to create a lipid model at a proper level of coarse-graining. Unfortunately, the two rules were not obeyed well when developing the existing models. For example, the united atom model [23] only omits the degrees of freedom of hydrogen atoms; it cannot improve the computation efficiency significantly. In the inverted Y-shape model, each hydrophobic tail bead contains three heavy atoms, but the choline, phosphate and glycerol groups (H and E head beads) contain six to eight heavy atoms. In the modified Y-shape model, the glycerol E bead is separated into three beads [23], but the corresponding types assigned to the beads are not consistent with the groups’ polarizabilities. The four-to-one MARTINI-like model obeys the mapping rules better. However, the DPD force field for this model, given in Ref. [31–33] was obtained based on the three-to-one mapping strategy. Therefore, it is essential to develop a new set of DPD force parameters for the MARTINI-like lipid model.

In practice, the DPD interaction parameters, *a*, are related to the liquid compressibility and solubility. These parameters are also dependent on the number of water molecules per DPD bead, *N*_*m*_. To reproduce the correct compressibility of water solvent at *N*_*m*_ = 4, a larger value of *a* = 100 is assigned to beads of the same type in this article. The repulsion parameters between beads of different type can be obtained by matching the Flory-Huggins *χ* parameter. To find the relationship between *a* and *χ* at *N*_*m*_ = 4, simulations of the mixture of two components at the present repulsion *a* = 100 between equal beads are performed first in this work. A new linear relation between *χ* and the excess of the mismatch repulsion is established. The repulsion parameters between the beads of phospholipids and water are calculated by matching the *χ* values available in the literature. Then, we carefully modify this parameter set to reproduce the structure and thermodynamic properties of the dimyristoyl phosphatidylcholine (DMPC), dipalmitoyl phosphatidylcholine (DPPC), and dioleyl phosphatidylcholine (DOPC) lipid bilayers, as well as to form vesicles. The membrane thickness, area per lipid, lipid orientation, bending rigidity, rupture behavior, and free energy for flip-flopping a lipid molecule in a bilayer obtained here demonstrate the validation of our new model. Hence, the paper is organized as follows. In Sec. II, we give a brief introduction to the DPD method. Then, we describe the force parameterization procedure for the h-shape lipid model based on a four-to-one CG mapping scheme and test the compatibility of the model by simulating DMPC, DPPC, and DOPC phospholipids bilayers and vesicles in Sec. III. The conclusions are given in Sec. IV.

## Methods

### DPD Simulation Methods

In the CG DPD simulation, the elementary unit is a soft bead with mass *m*_0_ and diameter *r*_0_ [8–10, 12]. For two beads with separation distance *r*_*ij*_ < *r*_0_, the beads interact via short-ranged repulsive forces

$$\begin{array}{c} F_{ij}^{C}\left(r_{ij}\right)=a_{ij}\left(1-r_{ij}/r_{0}\right){\hat{r}}_{ij}, \end{array}$$ (1)

random forces

$$\begin{array}{c} F_{ij}^{R}\left(r_{ij}\right)=\sqrt{2\gamma_{ij}k_{B}T}\left(1-r_{ij}/r_{0}\right)\zeta_{ij}{\hat{r}}_{ij}, \end{array}$$ (2)

and dissipative forces

$$\begin{array}{c} F_{ij}^{D}\left(r_{ij}\right)=-\gamma_{ij}{\left(1-r_{ij}/r_{0}\right)}^{2}\left({\hat{r}}_{ij}\cdot v_{ij}\right){\hat{r}}_{ij}. \end{array}$$ (3)

Here, the vectors **v** *_ij_* ≡ **v** *_i_*–**v** *_j_* are the velocity differences between particles *i* and *j*. The parameters *a*_*ij*_ (in unit of *k*_*B*_*T*/*r*_0_) are the repulsion strengths, γ_*ij*_ (in unit of $\sqrt{k_{B}Tm_{0}/r_{0}^{2}}$) are the friction coefficients, and *ζ*_*ij*_ are symmetrically and uniformly distributed random numbers.

If a molecule is a polymer, its bonds interact via harmonic potentials [12]

$$\begin{array}{c} E_{2}\left(r\right)=\frac{1}{2}K_{2}{\left(r-L_{0}\right)}^{2} \end{array}$$ (4)

with spring constant *K*_2_ and equilibrium length *L*_0_. The bond bending stiffness is described by

$$\begin{array}{c} E_{3}\left(r\right)=K_{3}\left[1-\text{cos}\left(\theta -\theta_{0}\right)\right] \end{array}$$ (5)

with force constant *K*_3_ and equilibrium angle *θ*_0_.

The equation of motion is integrated with a modified velocity Verlet algorithm with a time step 0.02*τ* ($\tau =r_{0}\sqrt{m_{0}/k_{B}T}$). DPD simulations are performed in either constant volume and constant temperature (NVT) or constant pressure and constant temperature (NPT) ensembles, combined with periodic boundary conditions.

### Simulation Setup

To prepare planar bilayer membranes, 1152 lipid molecules are placed in the center *X*−*Y* plane of an *L*_*x*_ × *L*_*y*_ × *L*_*z*_-sized box. The head groups are set on the outside of the membrane and the alkyl chains inside the membrane. The initial values of *L*_*x*_ = *L*_*y*_ are determined by using the desired projected area per lipid *a*_*prj*_, and *L*_*z*_ is set to 24*r*_0_. Water beads are distributed randomly in the space unoccupied by the membrane. The whole system has bead density $\rho =3/r_{0}^{3}$. To study the self-assembly of the vesicle, a same sized planar bilayer is placed in a bigger box such that the bilayer patch does not see the periodic boundaries. The size of the membrane and box selected here is big enough so that the finite size effects can be ignored, and small enough to ensure fast simulations [14].

### Analysis

#### Structural Properties of Bilayer

First, DPD simulations are performed in a constant normal pressure (Nγ_*s*_P_⊥_T) ensemble to obtain a relaxed planar bilayer with zero surface tension. Surface tension is a macroscopic quantity that is defined as the average of the difference between the normal and tangential pressure multiplied by the dimension of the simulation box in the direction normal to the bilayer,

$$\begin{array}{c} \gamma_{s}=\left\langle L_{z}\times \left[P_{z}-0.5\times \left(P_{x}+P_{y}\right)\right]\right\rangle . \end{array}$$ (6)

Here, we employ the Langevin piston approach [38] to maintain a constant normal pressure P_⊥_ and zero surface tension by adjusting the length of the simulation box perpendicular to the bilayer normal. The tensionless bilayers are used to analyze the membrane thickness *l*_*mem*_ (average distance between the choline groups in two leaflets), the area per lipid *a*_0_, and the orientation order of the alkyl tails *S*_*chain*_. Here, the orientation order is defined by

$$\begin{array}{c} S_{chain}=0.5\langle 3\cos^{2}\theta -1\rangle , \end{array}$$ (7)

where *θ* is the angle between the orientation of the vector along the hydrocarbon chain and the normal of the bilayer plan. The average is taken of the ensemble average over all lipids.

#### Elastic Properties of Bilayer

We also investigate the elastic response of the bilayer to a stress by measuring the membrane tension as a function of the projected area per lipid *a*_*prj*_. To mimic the stress, the area per lipid is varied by modifying the lateral size while keeping the number of lipid molecules and system volume fixed (in the NVT ensemble). Close to the tensionless state, the membrane tension Σ, as a function of the area per lipid, can be linearly fit by [39]

$$\begin{array}{c} \Sigma =K_{A}\left(a_{prj}-a_{0}\right)/a_{0}, \end{array}$$ (8)

where *K*_*A*_ is the area compressibility. The bending rigidity is calculated by [39]

$$\begin{array}{c} \kappa =K_{A}l_{mem}^{2}/48. \end{array}$$ (9)

This simple relation was obtained by fitting to the fluctuation spectrum [39]. Usually, it needs long simulation time and large membrane size to calculate the fluctuation spectrum. Here we use Eq (9) to estimate the bending rigidity.

#### Potential of Mean Force for Lipid Flip-Flop

The potential of mean force (PMF) for a lipid flip-flop in a bilayer is calculated by using the umbrella sampling method. The umbrella harmonic potential with a force constant of 200 *k*_*B*_*T* acts on the phosphate head bead of one lipid. We perform 61 simulations corresponding to 61 windows. In the first simulation, the constrained lipid is placed in the center of the bilayer. In the remaining 60 simulations, the lipid is shifted by 0.15*r*_0_ (approximately 0.1 nm) per simulation. The starting structures corresponding to the 61 windows are created by pulling one lipid from a well-structured bilayer to its window location using the umbrella potentials, with a lower force constant of 10 *k*_*B*_*T*, in a 10,000 time-steps simulation. Each window is then equilibrated for 50,000 time-steps with the full force constant, followed by a 50,000 time steps production simulation. The PMFs are calculated by using the weighted histogram analysis method (WHAM) [40].

## Results and Discussion

### Force Field Parameterization

In this work, we use the four-to-one mapping scheme of the MARTINI model [4–6, 29], i.e., a single CG site is composed of nearly four heavy atoms. Therefore, each bead has a mass and volume comparable to four water molecules. These mass and volume values are used to define the units of mass *m*_0_, length *r*_0_, and time *τ*. Here, *r*_0_ ≈ 0.71 nm and *τ* ≈ 143 ps in physical units according to this mapping. According to the functional group, a lipid molecule is modeled as a polymer connected by hydrophilic and hydrophobic beads, [4, 6, 10, 12, 14, 22, 23] as shown in Fig 1D. The beads are sorted into charged (Q), polar (P), nonpolar (N), and apolar (C) types. Each type is further divided into sublevels based on their hydrogen donor capacities (d), hydrogen acceptor capacities (a), and no hydrogen bond forming capacities (0).

In DPD simulations, the repulsion parameter *a*_*ii*_ is usually optimized to reproduce the compressibility *k*^−1^ of the system [9, 10], which is given by

$$\begin{array}{c} k^{-1}=1+2\alpha a_{ii}\rho /k_{B}T\left(\alpha =0.101\pm 0.001\right). \end{array}$$ (10)

For a pure water system, when the bead density *ρ* = 3, the choice of water-water repulsion *a*_*WW*_ = 100 can accurately reproduce the water compressibility *k*^−1^ ≈ 16 at room temperature [9, 10] (so it was assumed that the reduced temperature T* = 1 was corresponding to 25°C) and mapping number *N*_*m*_ = 4. For a multiple component system, the repulsion between beads of the same type is usually set equal to *a*_*WW*_ based on the assumption that the volume of each DPD bead is same for all species. (It is worth noting that when the constrain of constant volume is removed, a density mapping approach proposed by Ortiz et al [41] should be applied such that the bulk density of other pure species matches experimental data.) Other force parameters *a*_*ij*_ between beads of different types can be obtained from the relationship between the mutual solubility of polymers in water [9, 10], which is expressed by the Flory-Huggins *χ*-parameter and the excess repulsion Δ*a* = *a*_*ij*_−*a*_*WW*_,

$$\begin{array}{c} \chi =\lambda \Delta a. \end{array}$$ (11)

In this equation, the fitting parameter *λ* is dependent on the mapping number *N*_*m*_ (or the corresponding water-water repulsion *a*_*WW*_). For *N*_*m*_ = 1 and 3, *λ* have been shown having values of 0.286 ± 0.002 and 0.231 ± 0.001 at density *ρ* = 3 [9, 10]. For *N*_*m*_ = 4, it is necessary to redo the linear fitting. Neimark’s group also suggested another approach for determining the interaction parameters by fitting the parameters to the infinite dilution activity coefficient of binary solutions formed by reference compounds that represent CG fragments of surfactant molecules [34–37]. Here we use the method by fitting the force parameters to the Flory-Huggins paramenters [9, 10].

The *χ*-parameter represents the excess energy of the mixing of two components A and B in the Flory-Huggins models. If a cell is filled by a fraction *ϕ* of A-type beads and by a fraction 1−*ϕ* of B-type beads, and A and B beads occupy the same volume (*N*_*A*_ = *N*_*B*_ = *N*_*m*_), then the *χ*-parameter follows the equation

$$\begin{array}{c} \chi =\frac{\ln[(1-\phi )/\phi ]}{1-2\phi }. \end{array}$$ (12)

**Fig 2:**
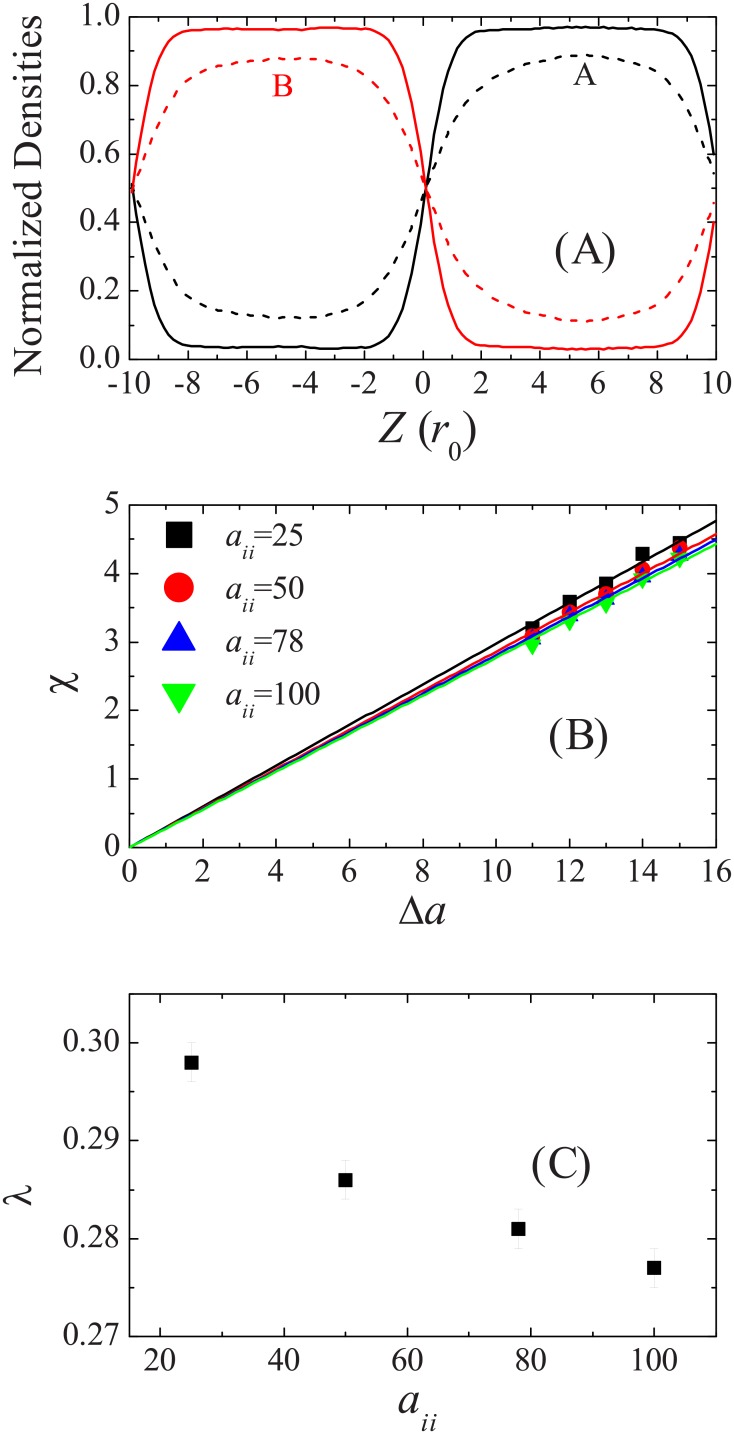
Relationship between Flory-Huggins parameters and repulsion parameters. (A): Normalized density profiles for bicomponent system at repulsion parameters *a*_*AA*_ = *A*_*BB*_ = 100 and *a*_*AB*_ = 110 (dashed lines) and *a*_*AB*_ = 113 (solid lines). (B): Relationship between excess repulsion and effective *χ*-parameter obtained at various *a*_*ii*_. The straight lines are the linear fittings to the data. (C): The constants of proportionality *λ* extracted from linear fitting in (B) at various *a*_*ii*_.

If *χ* is positive and exceeds a critical value, A-rich and B-rich domain segregation will take place. According to this equation, we perform DPD simulations of a bicomponent system composed of 3000 A-type beads and 3000 B-type beads in a box of size 10*r*_0_ × 10*r*_0_ × 20*r*_0_. In the initial condition, A-type beads are place in the upper half of the box, while B-type beads occupy the lower half of the box. The volume fractions *ϕ* are obtained for repulsion *a*_*AA*_ = *A*_*BB*_ = 100 and *a*_*AB*_ = 110 to 116. NVT simulations are performed for 2 × 10^5^ time steps. The first 10^5^ steps are used to equilibrate the system. Averages of the volume fraction of the last 10^5^ steps are taken. Typical normalized density profiles for the mixture, simulated at *a*_*AB*_ = 110 and 113, are given in Fig 2A. The mean value of *ϕ* over the slab, where the density is homogenous, is taken to compute the corresponding *χ*-parameter. The *χ*-parameter, as a function of the excess repulsion parameter Δ*a*, is plotted in Fig 2B. For comparison, we also present the results simulated for repulsions *a*_*AA*_ = *a*_*BB*_ = 25, 50, and 78 with *N*_*m*_ = 1, 2, and 3, respectively. Same results are also obtained if the simulation box has double length in Z-direction (data not shown). Good linear relations between *χ* and Δ*a* can be found, they are

$$\begin{array}{c} \chi =\left(0.298\pm 0.002\right)\Delta a\;\left(N_{m}=1\right), \end{array}$$ (13)

$$\begin{array}{c} \chi =\left(0.286\pm 0.002\right)\Delta a\;\left(N_{m}=2\right), \end{array}$$ (14)

$$\begin{array}{c} \chi =\left(0.281\pm 0.002\right)\Delta a\;\left(N_{m}=3\right), \end{array}$$ (15)

$$\begin{array}{c} \chi =\left(0.277\pm 0.002\right)\Delta a\;\left(N_{m}=4\right). \end{array}$$ (16)

The constant proportionality decreases when the mapping number *N*_*m*_ increases, but it is far from linear (Fig 2C). We note that *λ* = 0.231 ± 0.002 for *N*_*m*_ = 3 was given in Ref. [10], which is much smaller than the value we obtain here. From our simulations, we find that Δ*a* in the range from 4 to 8 (as used in Ref. [10]) was too small to give a rise to homogenous *ϕ* distribution. It can be seen from Fig 2A that even when Δ*a* = 10, it is hard to estimate the averaged volume fraction. Groot and Warren also claimed that there was good linear relation between *χ* and Δ*a* when *χ* > 3. We suppose that the small value of 0.231 given before [10] might be caused by a statistical error or just a print error.

Next we estimate the DPD repulsion parameter for a CG lipid in a water solution. The Flory-Huggins *χ*-parameter can be obtained from experiments or all-atom simulations [10]. The first *χ*-parameter is the interaction between hydrocarbon and water. Based on the experiments data of volume fraction for oil in water [42], Groot and Rabone derived *χ* = 6.0 for *N*_*m*_ = 3. The value 6.0/Nm = 2.0 is compared to that obtained by matching the critical micelle concentration of surfactant solutions [43]. Leermakers and Scheutjens used *χ*-parameter per carbon atom with value of 1.6 [44]. If the *χ*-parameter between hydrocarbon and water is determined by matching the solubility of water in oil [42], a higher value *χ*_*WC*_ = 9.3 at *N*_*m*_ = 3 [10, 45] or *χ*_*WC*_ = 12.4 at *N*_*m*_ = 4 can be obtained. According to Eq (16), the repulsion *a*_*WC*_ should be in the range from 123 to 145. Here, we set *a*_*WC*_ ≈ 130 based on *χ*_*WC*_ ≈ 8.0, as used by Groot and Rabone [10]. Our trial simulations show that if *χ*_*WC*_ has lower or higher value, for example, of 6.4 or 12.4, the compressibility and bending rigidity of the obtained lipid bilayer do not match the real membrane well. Following this strategy and using *χ*-values similar to those of Groot and Rabone’s work, we obtain all of the repulsion parameters *a*_*ij*_ (Table 1) based on the four-to-one mapping scheme. To reproduce the structure and elastic properties of the lipid bilayer membrane, some simplification and fine tuning of the force parameters are performed. For instance, we did not explicitly consider the charges between the zwitterionic head groups. To represent the electrostatic interactions, the repulsion between the same charged beads is increased. The force between the water and head group is set to less than *a*_*WW*_. The friction coefficients γ_*ij*_ are set to 4.5, 9, and 20 for low, mediate, and high repulsions, respectively [12].

**Table 1:** DPD force parameters *a*_*ij*_ (*k*_*B*_*T*/*r*_0_).

*a*_*ij*_	*W*	*Q*_0_	*Q*_*a*_	*N*_*a*_	*C*
*W*	100	98	98	102	130
*Q*_0_	98	110	100	102	130
*Q*_*a*_	98	100	110	102	130
*N*_*a*_	102	102	102	100	110
*C*	130	130	130	110	100

For bond interactions, the equilibrium CG bond lengths and angles and the respective force constants of lipids are obtained by fitting the bond distributions derived from AAMD simulations [27]. First, we simulate 16 DMPC lipids in a box containing 1600 water molecules using Amber force fields. In this concentration, the DMPC lipids do not form an ordered structure. We then calculate the bond and angle distributions of the center of masses of the CG beads. The distributions are fitted by Gaussian functions [27]

$$\begin{array}{c} P\left(\theta \right)=\frac{A}{w\sqrt{\pi /2}}\exp^{-2{\left(\theta -\theta_{c}\right)}^{2}/w^{2}}. \end{array}$$ (17)

Here, the structure parameter *θ* can be a bond or an angle. The fitting parameter *θ*_*c*_ is the distribution center, *A* is the area, and *w* is the width, which is related to the force constant by *K*_2_ (*or* *K*_3_) = 4*k*_*B*_*T*/*w*^2^. The corresponding bond interaction potentials can be obtained by Boltzmann inversion of Eq (17). Fits of the *C*−*C* bond and *C*−*C*−*C* angle distributions of the DMPC molecules give *L*_*CC*_ ≈ 0.47*nm* ≈ 0.66*r*_0_, $K_{2}\approx 512k_{B}T/r_{0}^{2}$, *θ*_0_ ≈ 174°, and *K*_3_ ≈ 6*k*_*B*_*T*. In our simulations, the two connected beads interact via both DPD repulsion and harmonic bond interaction; thus, the bond length parameters *L*_0_ in Eq (4) are set to a small value of *L*_0_ ≈ 0.59*r*_0_ to achieve the fitted equilibrium length. For lipids in a bilayer, the hydrocarbon tails are more compacted; thus, we choose *θ*_0_ to be 1800° for *C*−*C*−*C* angles. All of the bond parameters for DMPC are given in Table 2. For DPPC lipid, each of its tail has two more CH_2_ groups than DMPC, thus is modeled by four connected *C*-type beads. Each CG DOPC lipid tail is also composed of 4 beads, but the *C*−*C*−*C*_*T*_ (*C*_*T*_ stands for the terminal tail bead) angle of both tails is set to *θ*_0_ ≈ 120° to mimic the unsaturated hydrocarbon chains.

**Table 2:** Equilibrium bond lengths, angles, and force parameters for DMPC lipid.

bond	*L*_0_ (*r*_0_)	$K_{2}\;\left(k_{B}T/r_{0}^{2}\right)$	angle	*θ*_0_ (degree)	*K*_3_ (*k*_*B*_*T*)
1-2	0.47	512	2-3-4	180	6
2-3	0.47	512	2-3-7	120	6
3-7	0.31	512	3-4-5	180	6
3-4	0.59	512	4-5-6	180	6
4-5	0.59	512	7-8-9	180	6
5-6	0.59	512	8-9-10	180	6
7-8	0.59	512			
8-9	0.59	512			
9-10	0.59	512			

### Bilayer Properties

**Fig 3:**
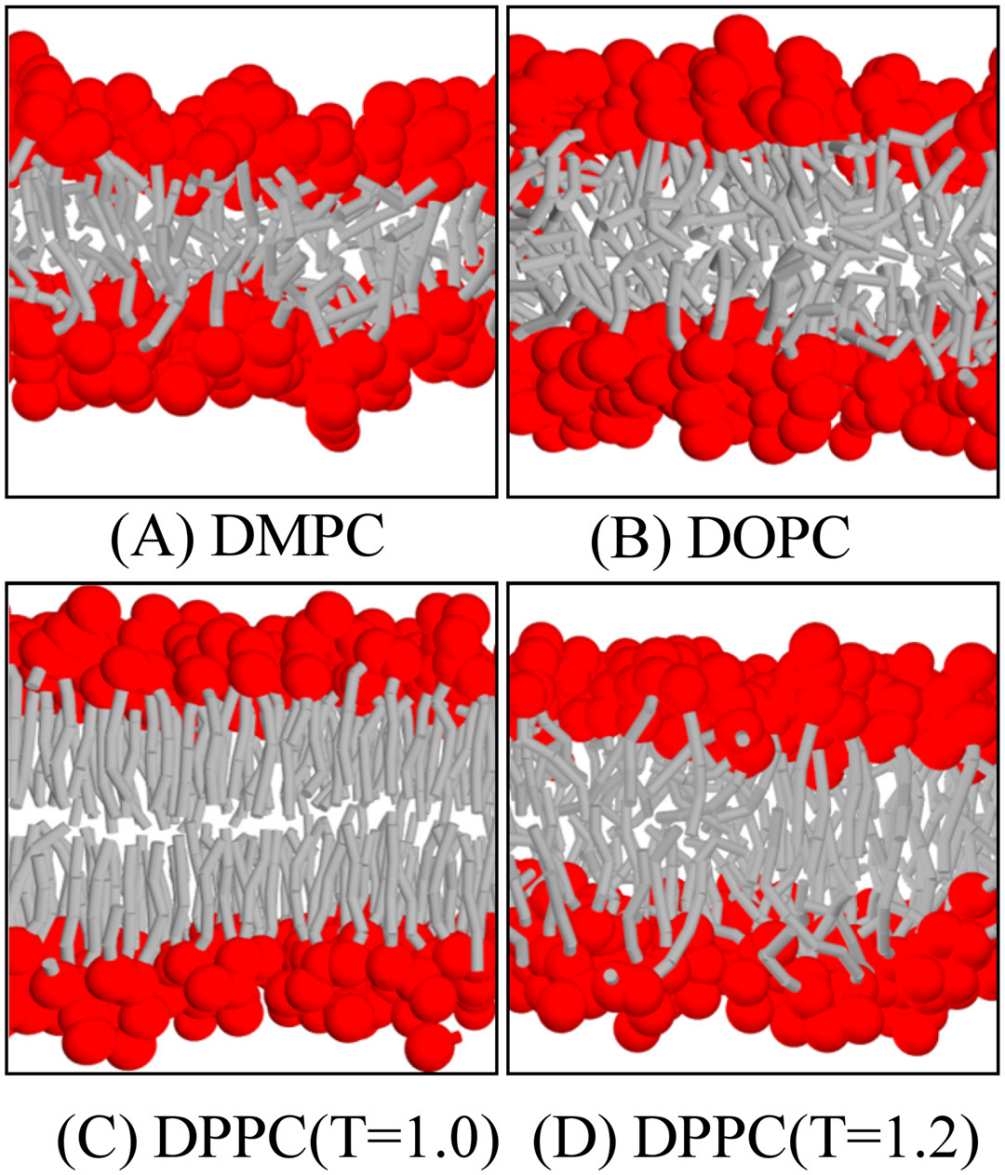
Snapshots of (A) DMPC, (B) DOPC, (C) gel-phase DPPC, and (D) fluid-phase bilayers at zero tension states.

**Fig 4:**
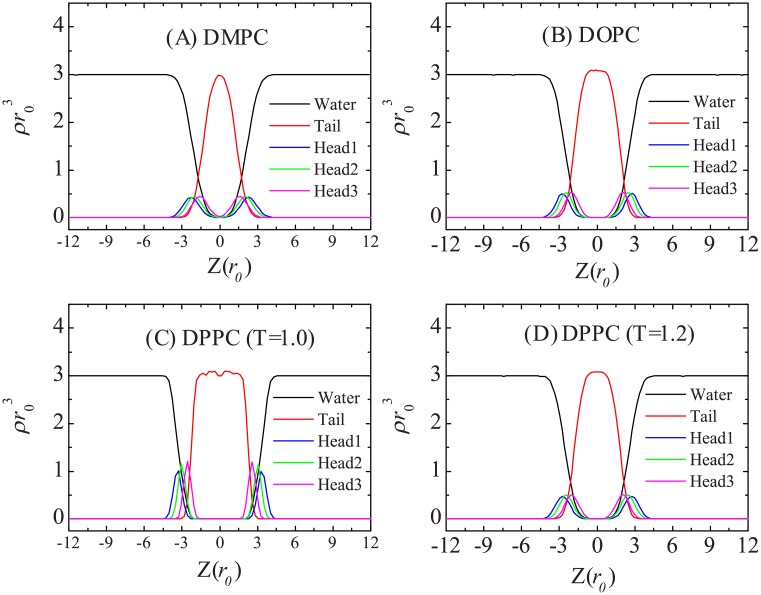
Density profiles of (A) DMPC, (B) DOPC, (C) gel-phase DPPC, and (D) fluid-phase DPPC bilayers at zero tension states.

We first simulate pure DMPC, DOPC, and DPPC lipid bilayers (each bilayer is composed of 1152 lipids) in the Nγ_*s*_P_⊥_T ensemble at zero surface tension and temperature *T* = T* = 1. Here, the normal pressure of P_⊥_ is set to 89 $k_{B}T/r_{0}^{3}$, which is same to the pressure for bulk water simulated in NVT ensemble at density $\rho =3/r_{0}^{3}$ and mapping number *N*_*m*_ = 4. Figs 3 and 4 give the snapshots and density distribution profiles of these membranes, respectively. The membrane thickness *l*_*mem*_, the area per lipid *a*_0_, and the orientation order of the alkyl tails *S*_*chain*_ of these phospholipids bilayers are listed in Table 3. All of these structural properties are in good agreement with the experimental measurements [46]. The orientation order demonstrates that DMPC and DOPC bilayers are in the fluid phase, while the DPPC bilayer is in the gel phase at temperature T* = 1. We then increase the temperature to T = 1.2 for DPPC and find that the bilayer transitions to the fluid phase and have similar properties as DOPC bilayer. (It is should be noted that for the DPPC bilayer simulated at temperature T = 1.2, we still use the force parameters obtained at T = 1; it could be more precise if temperature-dependent force parameters were used [47, 48]). The snapshots of these membrane show that for the bilayer in a fluid phase, the hydrophobic tails are more spread out. The terminal tail beads belonging to opposing leaflets can even make contact with each other. This is reflected in the density profile where the curve of the tail bead is flat (for DOPC and DPPC at high temperature) or even has a peak (for DMPC) at the bilayer mid-plane. For the DPPC bilayer in the gel phase, the two leaflets are well separated, with their terminal tail beads out of reach. The corresponding density profile of the tail beads has a minimum at the bilayer mid-plane. The density profiles also indicate that in the gel-phase bilayer, the hydrophobic core is more confined such that water hardly penetrates into the membrane.

**Table 3:** Structural properties of phospholipids bilayers: membrane thickness *L*_*mem*_, area per lipid *a*_0_, and orientation order of the hydrocarbon chain *S*_*chain*_ as well as elastic properties: bending rigidity *κ* and rupture tension Σ_*r*_.

lipid	*L*_*mem*_ (nm)	*a*_0_ (nm^2^)	*S*_*chain*_	*κ* (10^−19^ J)	Σ_*r*_ (mN/m)
DMPC(T = 1)	3.56	0.66	0.47	0.5	4
DOPC(T = 1)	3.97	0.70	0.45	0.6	5
DPPC(T = 1)	4.73	0.58	0.88	11.0	5
DPPC(T = 1.2)	3.99	0.69	0.54	0.6	5

**Fig 5:**
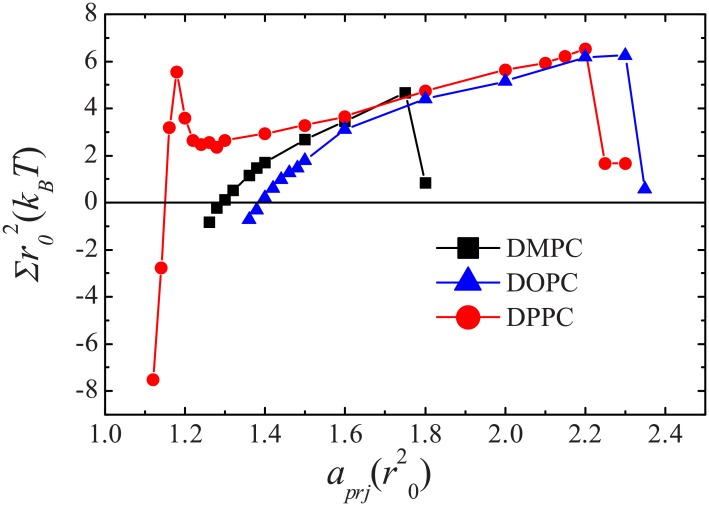
Membrane tension Σ as a function of the projected area per lipid *a*_*prj*_ for DMPC, DOPC, and gel-phase DPPC bilayers.

**Fig 6:**
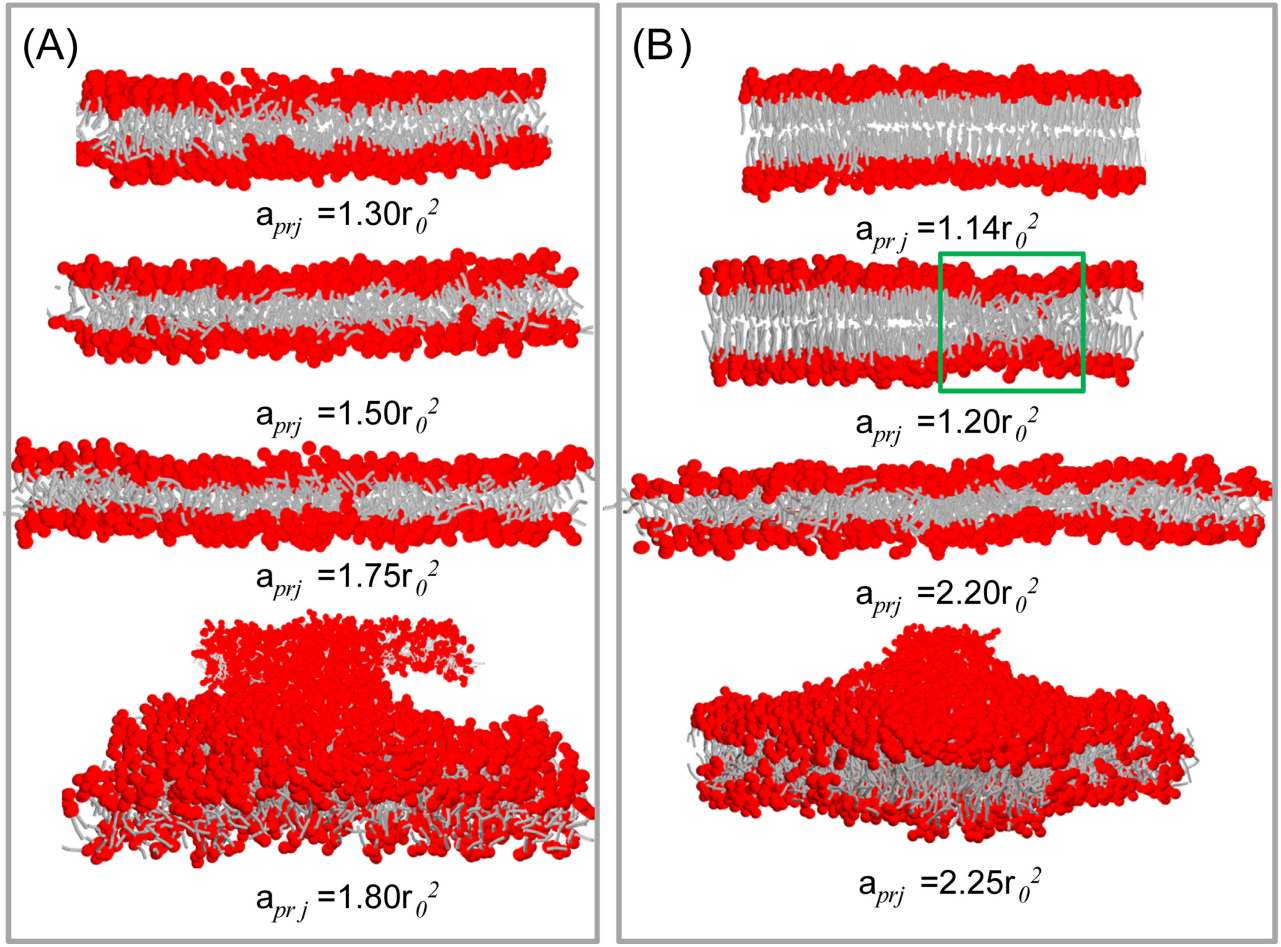
Snapshots of (A) DMPC and (B) DPPC (simulated at temperature *T* = 1) bilayers under stretching.

The elastic responses of various lipid bilayers to a stress are given in Fig 5 and Table 3. We find that the DMPC membrane is in the tensionless state at $a_{0}\approx 1.30r_{0}^{2}\approx 0.66$ nm^2^; this is the same result obtained in the Nγ_*s*_P_⊥_T ensemble. Then, the membrane becomes thin and tense upon stretching. The bilayer ruptures when its area is stretched by approximately 40%; see Fig 6A. The rupture tension is close to $5k_{B}T/r_{0}^{2}\approx 4$ mN/m. The area compressibility *K*_*A*_ and the bending rigidity *κ* for the DMPC bilayer are approximately $23k_{B}T/r_{0}^{2}\approx 188$ dyn/cm and 11*k*_*B*_*T* ≈ 0.5 × 10^−19^ J, respectively. These mechanical properties are also comparable to the experimental data [49]. For the DOPC bilayer, the tensionless state shifts to $a_{0}\approx 1.39r_{0}^{2}\approx 0.70$ nm^2^. Because the DOPC bilayer is thicker than the DMPC bilayer, it is more stretchable and can resist a stretch of 60%. The corresponding rupture tension is approximately 5 mN/m. At the same time, the bending rigidity of the DOPC membrane is up to *κ* ≈ 14*k*_*B*_*T*.

The gel-phase DPPC bilayer exhibits different elastic response to a stress (Fig 5). Close to the tensionless state, only a 3% stretching can induce a tension of $5k_{B}T/r_{0}^{2}$ in the DPPC bilayer, which is already enough to trigger rupture of the DMPC and DOPC bilayers. However, the DPPC bilayer does not rupture at this tension at all. More surprisingly, the induced tension actually decreases when the area of the DPPC bilayer is further stretched by up to 10%. Upon more stretching, the DPPC bilayer presents similar elastic response as the DOPC bilayer. We find that the strange elastic response of the gel-phase membrane is caused by the phase transition induced by the stress, as illustrated by the snapshots in Fig 6B. The lipids in the gel phase are very compacted; thus, it costs more energy to stretch its area. The sharp increase of the tension reflects such behavior. Upon more stress [(*a*_*prj*_−*a*_0_)/*a*_0_ in the range of 3% to 10%], some lipids tilt their tails to prevent the penetration of water into the hydrophobic core of the membrane, Fig 6B. This results in localized fluid-phase domains in the bulk gel-phase. The lipids in the fluid phase are less compact and relatively easier to be stretch than those lipids in the gel-phase; thus, the tension curve starts to decrease. When the membrane area is stretched by more than 10%, the DPPC bilayer transits to a pure fluid-phase (Fig 6B) and expresses normal elastic properties.

**Fig 7:**
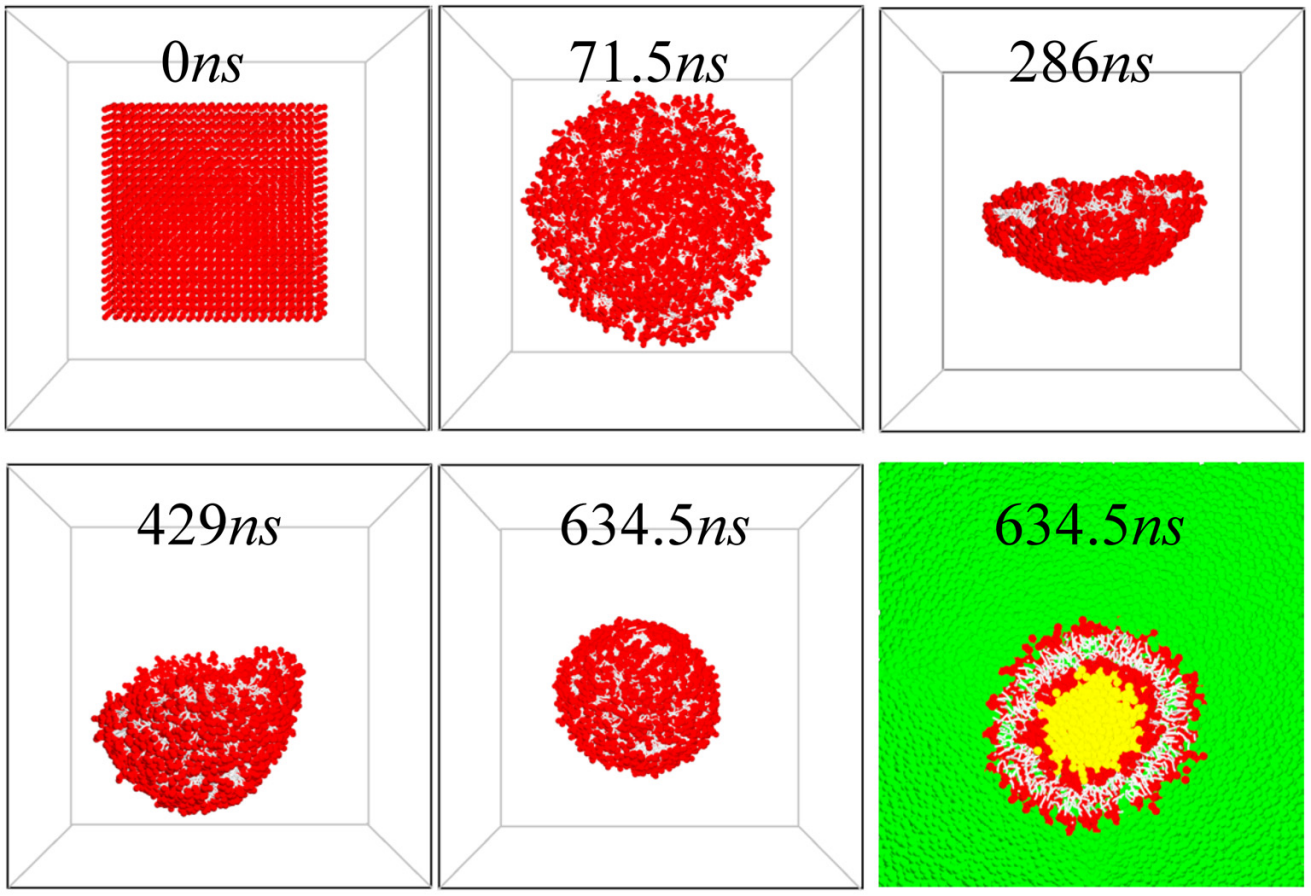
Snapshots of the evolution of DMPC vesicle formation. The last snapshot is the cross-sectional view of a cut vesicle, with explicit inner (yellow) and outer (green) water.

We also simulate the self-assembly of the fluid-phase vesicle, starting from a planar bilayer configuration. The snapshots in Fig 7 show the evolution of DMPC vesicle formation. The bilayer first shrinks to a round bicelle shape, then wraps up and closes into a vesicle. Once a vesicle forms, water leakage rarely occurs, which indicates that the vesicle is well-sealed. DOPC bilayers self-assemble into vesicles in the same manner (snapshots not shown). Whether a vesicle can form is determined by the bending properties of the membrane. These results demonstrate that our model and the corresponding force parameters are good enough to reproduce the correct bending rigidity (in the range of 10 to 20 *k*_*B*_*T*) of the membranes.

**Fig 8:**
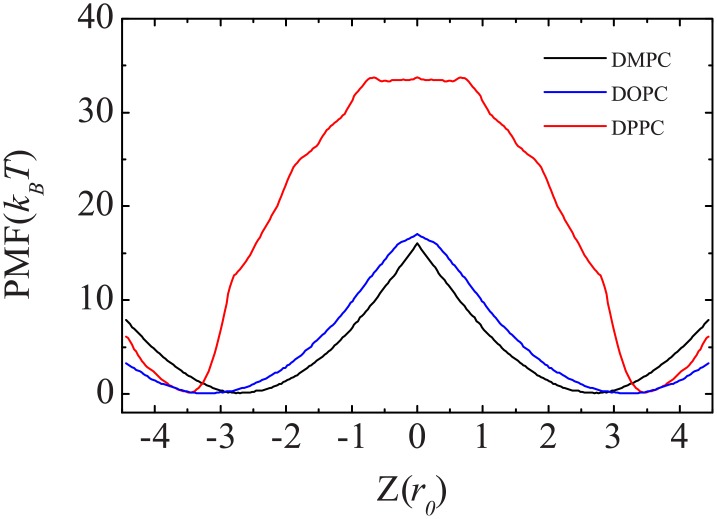
Potential of mean forces for DMPC, DOPC, and DPPC lipid flip-flop in a DMPC, DOPC, and DPPC bilayer, respectively.

Another thermodynamic quantity we measured is the free energy cost for transferring a lipid from equilibrium to the bilayer center by using the umbrella sampling. As shown in Fig 8, for fluid-phase DMPC and DOPC bilayers, there is a free energy minimum at the equilibrium position and a steep slope as the head group moves into the bilayer center. The free energy barrier is approximately 16 *k*_*B*_*T* (or 40 kJ/mol) for DMPC lipid flip-flop and slightly larger (approximately 17 *k*_*B*_*T*) for DOPC lipid flip-flop. Moving the lipid from its equilibrium position into the solvent also has a large free energy cost. For a gel-phase DPPC bilayer, it cost 34 *k*_*B*_*T* (or 85 kJ/mol) to flip-flop a lipid. The free energy cost for the lipids of DMPC and DPPC are similar to the barriers obtained from AAMD and CG MARTINI simulations, but the energy for DOPC is lower than that AAMD simulation [50, 51]. The PMF for DPPC flip-flops plateaus near the bilayer center. This implies that at the bilayer center, the DPPC lipid is in a locally homogeneous environment, i.e., it is not interacting with the leaflets at the water-lipid interface. In contrast, the steep slope of the PMF at the bilayer center for DMPC and DOPC lipids indicates that even when the head group of the lipid is at the center of the bilayer, the lipid is still making contact with one leaflet of the bilayer.

**Fig 9:**
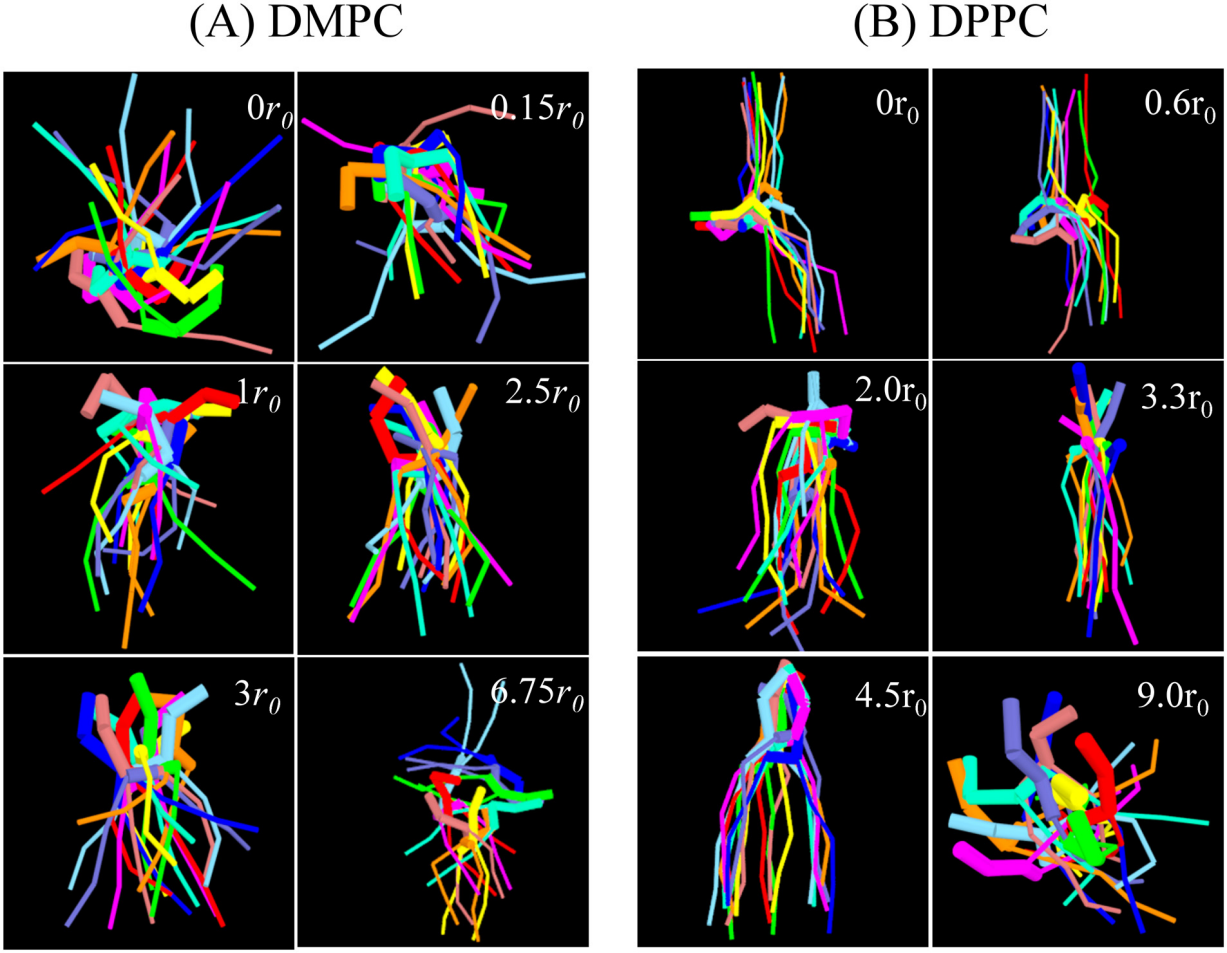
Snapshots of (A) a constrained DMPC lipid and (B) a DPPC lipid at different locations relative to the center of the membrane. Each color represents the configuration of the lipid at a different time, taken from the last 5 × 10^5^ time steps and separated by 5 × 10^4^ time steps. The overlaying of the configurations illustrates the flexibility and orientation of the constrained lipid molecule. Head groups and tails of the lipid are represented by thick and thin bonds, respectively.

The configurations of lipids at different locations relative to the center of the membrane in Fig 9 show that in the equilibrium position (2.5 *r*_0_ for DMPC and 3.3 *r*_0_ for DPPC), the lipid is more compact and has the same elongated orientation as the surrounding lipids. As the lipid is pulled into the bilayer and solvent, it adopts a broad range of orientations. The lipid adopts these variant orientations to match the hydrophilic environment. These observations are similar to the AAMD results [50, 51]. DMPC and DPPC lipids exhibit similar conformation distributions near their equilibrium positions even though they are in different phase bilayers. However, distinctions appear at the center of the membrane. At this position, the heads of a DPPC lipid change their orientation and become almost perpendicular to the membrane normal, while the two tails splay dramatically and point to opposite directions along the bilayer normal. In contrast, the DMPC lipid at the bilayer center already flip-flopped and has an averaged orientation along the bilayer normal. This means that the lipid is still interacting with the hydrophilic head groups of one leaflet of the bilayer and water. As a matter of fact, the snapshots at *r* = 0 and *r* = 0.15*r*_0_ in Fig 9A indicate that the flip-flop of the DMPC lipid in a DMPC bilayer is transient. Once the flip-flop occurs, the lipid interacts with the opposite leaflet. We propose that the thickness and capacity of the membrane affect the lipid orientation. Because the terminal tail beads belonging to opposite leaflets can overlap in the fluid-phase membrane, it costs more energy to restrain the head of a lipid at the bilayer center. This property is reflected by the steep slope of the PMFs at the bilayer center for DMPC and DOPC lipids. Nevertheless, because the fluid-phase membrane is thin and less compact, the overall barrier for lipid flip-flop here is relatively low compared to that of the gel-phase bilayer. The thin membrane also allows the restrained lipid to change its orientation in a short time and interact with the membrane/solvent interface. On the other hand, the void at the bilayer center of the gel-phase membrane allows the restrained lipid to cross this region without energy cost. To minimize the membrane defect, the trapped lipid orientates its head to be perpendicular to the membrane normal but its tails to splay and be along the direction of the membrane normal. Because the gel-phase membrane is thicker and more compact than the fluid-phase membrane, the overall flip-flop barrier is higher.

As the lipid is pulled into the bilayer, a few water and lipid head groups may follow the movement of the lipid and form a defect. In AAMD simulation, the defect is followed by a water pore spanning the bilayer [50, 51]. However, the water defect or pore is not observed in our DPD simulations. As in the CG MARTINI method, DPD water has no dipole or polarizability; thus, the interaction of polar molecules might be underestimated in hydrophobic environments [50, 51]. The different flip-flop barriers obtained from AAMD and DPD simulations for DOPC lipid might be caused by the dipole effects. The lack of polarizability may also explain the configuration differences of a DPPC lipid at the bilayer center observed in the AAMD and DPD simulations: in AAMD simulations, the water pore promotes the lipid tail to adapt more random orientations; while the lack of a water pore in DPD simulations compels the lipid to splay its tail to minimize the contact between the hydrophobic tails of the bulk lipids and hydrophilic head groups of the restrained lipid. Coarse-grained model based on electrostatic multipole with implementation of DPD algorithm might be a solution to involve the lipid polarizability [52].

## Conclusion

Due to its soft potentials, coarse-grained DPD modeling allows for simulations of large systems and at longer time scales. In this article, we present a four-to-one CG mapping scheme of a phospholipid. This mapping ensures that the interaction units have similar mass and volume and well-defined physicochemical functions. A new set of optimized DPD force field parameters for the lipid molecules and water is derived. Our simulations show that this force field can accurately reproduce the structural properties of DMPC, DOPC, and DPPC lipid bilayers. Importantly, the elastic properties, such as the bending rigidity and rupture tension, of the bilayer obtained here are comparable to real biomembranes. The potential of mean force of transferring a lipid from its equilibrium position to the bilayer center is also in agreement with AAMD simulation. The effects of dissipation and thermal fluctuation accurately included in the DPD mode also compensate the lack of hydrodynamic behavior in CGMD models. All of these results indicate that the DPD parameters presented here make DPD a competitive approach for modeling biological materials.
